# In Silico Prediction and Analysis of Unusual Lantibiotic Resistance Operons in the Genus *Corynebacterium*

**DOI:** 10.3390/microorganisms9030646

**Published:** 2021-03-19

**Authors:** Oliver Goldbeck, Dominik Weixler, Bernhard J. Eikmanns, Christian U. Riedel

**Affiliations:** Institute of Microbiology and Biotechnology, Ulm University, 89081 Ulm, Germany; dominik.weixler@uni-ulm.de (D.W.); bernhard.eikmanns@uni-ulm.de (B.J.E.)

**Keywords:** *Corynebacterium*, antimicrobial peptides, bacteriocins, lantibiotics, resistance, nisin, in silico analysis

## Abstract

Post-translationally modified, (methyl-)lanthionine-containing peptides are produced by several Gram-positive bacteria. These so-called lantibiotics have potent activity against various bacterial pathogens including multidrug-resistant strains and are thus discussed as alternatives to antibiotics. Several naturally occurring mechanisms of resistance against lantibiotics have been described for bacteria, including cell envelope modifications, ABC-transporters, lipoproteins and peptidases. *Corynebacterium* species are widespread in nature and comprise important pathogens, commensals as well as environmentally and biotechnologically relevant species. Yet, little is known about lantibiotic biosynthesis and resistance in this genus. Here, we present a comprehensive in silico prediction of lantibiotic resistance traits in this important group of Gram-positive bacteria. Our analyses suggest that enzymes for cell envelope modification, peptidases as well as ABC-transporters involved in peptide resistance are widely distributed in the genus. Based on our predictions, we analyzed the susceptibility of six *Corynebacterium* species to nisin and found that those without dedicated resistance traits are more susceptible and unable to adapt to higher concentrations. In addition, we were able to identify lantibiotic resistance operons encoding for peptidases, ABC-transporters and two-component systems with an unusual predicted structure that are conserved in the genus *Corynebacterium*. Heterologous expression shows that these operons indeed confer resistance to the lantibiotic nisin.

## 1. Introduction

The genus *Corynebacterium* comprises a diverse set of Gram-positive, aerobic actinobacteria widely distributed in nature. Many species are commensals of the microbiome of humans and animals [1]. Besides biotechnologically relevant *Corynebacterium* species like *C. glutamicum* and *C. ammoniagenes* [2,3], the most prominent members of the genus are the toxicogenic human pathogen *C. diphtheriae* [4] and zoonotic *C. ulcerans* [5]. Both are causative agents of diphtheria, a disease which is nearly eradicated in developed countries but an ongoing problem in countries without efficient vaccination programs [6]. Less prominent are non-diphtheric pathogenic *Corynebacterium* species including the clinically relevant species *C. striatum*, *C. amycolatum*, *C. propinquum*, *C. minutissimum*, *C. pseudodiphthericum*, *C. urealyticum*, *C. aurimucosum* and *C. xerosis* [7]. These organisms are increasingly detected in respiratory and urinary tract infections, endocarditis, osteomyelitis, hospital-related infections of prosthetics, septic arthritis, peritonitis, bacteremia, meningitis and wound infections posing a threat to immune-compromised patients [8,9,10,11].

Although most *Corynebacterium* species are susceptible to antimicrobial therapy, there are recent reports of multi-resistant strains of e.g., *C. striatum*, *C. amycolatum* or *C. jeikeium* [9,12,13,14,15]. As for other multi-resistant pathogens, over- and misuse of antibiotics in human therapy, animal farming and food production appear to be drivers for their development [16]. Therefore, alternatives for antibiotics are needed.

Promising, but compared to standard antibiotics largely unexploited candidates are so called bacteriocins, which are ribosomally synthesized antimicrobial peptides produced naturally by many bacteria [17]. Over the years, different classes of bacteriocins have been discovered, including small, post-translationally modified (Class I) and unmodified (Class II), heat stable peptides as well as larger, heat-labile peptides and small proteins (Class III). A major subgroup within Class I are (methyl-)lanthionine-containing peptides, which contain dehydrated amino acids like dehydrobutyrine and/or dehydroalanine and are characterized by thioether bridges, so called lanthionine rings [18]. These lantibiotics, especially their most prominent member nisin, have been extensively studied for decades and are discussed as alternatives for antibiotics [19]. Nisin is one of the few bacteriocins approved by the U.S. Food and Drug Administration for human consumption, widely used in food preservation, and has potential for medical use [20].

Most lantibiotics share a similar, dual mode of action. Due to their cationic nature, they are attracted to the negatively charged surface of bacterial cells. Nisin and other lantibiotics then bind to the cell wall precursor lipid II, which is located in the outer leaflet of the cell membrane [21]. In consequence, lipid II is sequestered and peptidoglycan synthesis is inhibited [22]. At higher concentrations, pores consisting of lantibiotic/lipid II heterocomplexes are formed. This dual mode of action as well as the potent activity at nanomolar concentrations make lantibiotics attractive candidates for a wide range of medical applications.

In their natural habitat, bacteria produce lantibiotics and other bacteriocins to inhibit competitors and to establish themselves in their ecological niche [23]. Consequently, protective mechanisms have evolved. Producer strains possess dedicated immunity systems against their own bacteriocins. For example, nisin-producing *Lactococcus lactis* species harbor a nisin-binding lipoprotein NisI and the efflux ABC-transporter NisFEG that effectively protect cells against the produced lantibiotic [24,25].

Complementary to these immunity systems, many producer and non-producer species possess innate resistance mechanisms that are not specific for lantibiotics. Cell wall and membrane modification systems that introduce positive charges into the cell envelope build a first line of non-specific defense to repel a broad spectrum of cationic antimicrobial peptides (AMPs) [26]. For example, D-alanylation of wall and lipoteichoic acids via enzymes encoded by *dlt* operons is conserved across a wide range of species including pathogenic *Listeria monocytogenes*, *Staphylococcus aureus* and *Streptococcus agalactiae* [27]. Another mechanism to mask the negatively charged surface is aminoacetylation of phosphatidylglycerol (PG) in the cell membrane, by e. g. lysinylation generating lysinylated PG (L-PG) [28]. L-PG is found in numerous species and genes for phosphatidylglycerol lysyltransferases are conserved in many Gram-positive and Gram-negative genera [29]. The best studied examples are the multiple peptide resistance factor MprF in *S. aureus* and *B. subtilis* as well as the bifunctional protein LysX from *Mycobacterium tuberculosis*. These enzymes have been shown to significantly influence the susceptibility of the host organisms to nisin and other cationic antimicrobials like defensins or lipopeptides [30,31,32]. 

A second line of defense against AMPs is conferred by two and three-component regulatory systems and ABC-transporters that are often functionally and genetically connected [26]. In general, two types of ABC-transporters prevail in many Firmicutes species that both consist of at least one transmembrane (TMD) and a nucleotide binding domain (NBD). The cationic peptide resistance ABC transporter (CprABC-type) contains the domains CprA (NBD), CprB (TMD) and CprC (TMD) that assemble to the functional transporter. The best studied examples of this type are NisFEG of nisin-producing *L. lactis* and the eponymous CprABC of *Clostridioides difficile* strains [24,33]. The second type of ABC-transporters associated with lantibiotic resistance is the BceAB-type, named after the bacitracin efflux transporter of *B. subtilis*. They confer resistance to bacitracin but also other peptides like nisin or gallidermin [34,35]. BceAB-type transporters belong to the Pep7E-family of ABC-transporters and consist of a dimerizing NBD and a single TMD with 10 transmembrane-helices (TMH). A major difference between the two transporter families is the characteristic extracellular domain (ECD) between TMH 7 and 8 found in the BceAB-type. Prominent examples of BceAB-type transporters involved in lantibiotic resistance of pathogenic bacteria are VraDE of *S. aureus* or NsrFP of *S. agalactiae* [36,37].

Both, CprABC and BceAB transporters, have coevolved with conserved two-component systems (TCS) consisting of a histidine kinase (HK) and response regulator (RR) [38,39]. The TCS are functionally and genetically intertwined with the ABC-transporters and involved in sensing of the peptide substrate as well as in regulation of their expression. In case of CprABC, the HK CprK consists of two TMHs and an extracellular loop that is assumed to be involved in sensing of lantibiotics comparable to NisK, LanK and others [40]. By contrast, BceS and other HKs of BceAB-RS systems do not contain an ECD but directly interact with the BceB subunit of the transporter [35]. The TCS and ABC-transporters are often organized in conserved operons that may additionally comprise lipoproteins like NisI or lantibiotic degrading S41-peptidases such as the nisin resistance protein NSR [25,41].

The world-wide spread of antimicrobial resistance is a threat to treatments of an increasing number of infections caused by pathogenic bacteria, including *Corynebacterium* species. Lantibiotics that are active against these bacteria are a potential solution to this threat. With this in mind, resistance mechanisms amongst pathogenic, environmental and commensal bacteria have to be considered. To our knowledge, there is no comprehensive report on the susceptibility of the genus *Corynebacterium* to lantibiotics and the associated mechanisms despite its prominent position in the microbiome of humans and animals. Here, we report a bioinformatic analysis of lantibiotic resistance mechanisms in the genus *Corynebacterium* and suggest the presence of resistance operons encoding for lantibiotic degrading peptidases, ABC transporters and HKs with unusual structures and a potentially so far undescribed mechanism.

## 2. Materials and Methods

### 2.1. Strains, Plasmids and Oligonucleotides

All strains and plasmids are listed in Table 1; oligonucleotides are listed in Table S3.

### 2.2. BLASTP Analyses

The genome accession numbers of the *Corynebacterium* genomes available in the NCBI database (version of April 2020) that were analyzed using BLASTP [48] are listed in Table S1. Amino acid sequences of the proteins listed in Table S2 were retrieved from the UniProt database and were used as BLASTP query using default parameters (BLOSUM62; gap existence costs: 11; gap extension costs: 1). Each set of results was checked for duplicates and only hits with an e-value of <1e^−10^ were retained and further analyzed. To identify HK, RR, NBD and NSR proteins, neighboring genes were analyzed based on PFAM domain annotations or sequence similarity and added to the data set manually if not already present. Proteins with FtsX-like PFAM domains (PF02687) that are typically found in BceB subunits [38] were regarded as ABC-transporter permeases while proteins with S41-peptidase domain (PF03572) were regarded as NSR-homologs [41].

Multiple sequence alignments were conducted with the online tool MAFFT [49] and visualized using Jalview [50]. Prediction of transmembrane domains and their topology was carried out by TMHMM2.0 [51] and Protter [52].

### 2.3. Prediction and Analysis of Bacteriocin Gene Clusters

Prediction of bacteriocin gene clusters (BGCs) was carried out with BAGEL4 [53] using available genomic sequences of the species listed in Table S1. Areas of interest were further analyzed based on gene annotation and subdivided into three bacteriocin classes. To identify nisin-like bacteriocins, the deposited amino acid sequence of nisin Z (UniProt identifier P29559) was used as a query for BLASTP analysis of the *Corynebacterium* genomes with the above described parameters, resulting in four hits that were added to the dataset if not already present. BGCs were further analyzed for genes encoding resistance/immunity proteins and checked if they match with the above described BLASTP data set which was not the case for any of the BGCs.

### 2.4. Phylogenetic Analyses of Corynebacterium Species

16S rRNA gene sequences of the *Corynebacterium* species listed in Table S1 were acquired from EzBioCloud [54] and LPSN [55] as of January 2021. The *Rhodococcus hoagii* 16S rRNA gene sequence was selected as root for the phylogenetic tree. All sequences were collated using the free MEGA software (version 10.1.8) and aligned with the implemented MUSCLE [56] multiple sequence alignment tool. A neighbor-joining tree was generated using bootstrap replication values of 1000. The resulting tree was then graphically adjusted using the web tool iTol [57].

### 2.5. Molecular Biology Procedures

Cloning of plasmids for expression of lantibiotic resistance operons was conducted with standard reagents and according to the manufacturer’s protocol. All oligonucleotides listed in Table S3 were obtained from Eurofins Genomics (Ebersberg, Germany). Polymerase chain reaction (PCR) was performed with nucleotides from Bio-Budget (Krefeld, Germany) and Q5 high fidelity polymerase (New England Biolabs, Ipswich, USA) in a C100 thermocycler (Bio-Rad Laboratories, Munich, Germany). Genes of interest were amplified using primers introducing overlapping ends for subsequent Gibson Assembly^®^ [58] into pJC1. The empty vector was linearized by restriction endonucleases *Bam*HI and *Sal*I. All resulting plasmids are listed in Table 1. After verification of the assembly by Sanger sequencing by a commercial service provider (Eurofins Genomics, Ebersberg, Germany), plasmids were used to transform competent *C. glutamicum* ATCC 13032 cells by electroporation [59].

### 2.6. Determination of Minimal Inhibitory Concentration (MIC)

Dose-response experiments to determine MIC values were conducted using a microtiter plate dilution assay [60]. All bacterial strains listed in Table 1 were cultivated in 2xTY medium [61]. For strains carrying pJC1-derived plasmids, kanamycin (50 µg/mL) was added. Bacteria were streaked on 2xTY agar and incubated for 3 days at 30 °C. A single colony was then used to inoculate 5 mL 2xTY medium in glass tubes and cultivated overnight at 30 °C and aeration on an orbital shaker (130 rpm). The next day, fresh tubes with 5 mL 2xTY medium were inoculated with 100 µL of the overnight culture. In some experiments, nisin (Sigma-Aldrich, St Louis, MO, USA) was added to a final concentration of 0.25 µg/mL active compound for induction of potential resistance mechanisms. For *C. efficiens*, nisin concentration was reduced to 0.1 µg/mL for induction due to its higher sensitivity towards this AMP. The cultures were then grown at 30 °C with aeration for 3–5 h. After determination of the optical density at 600 nm (OD_600_), the cultures were diluted in fresh 2xTY and used in a 96-well plate (Sarstedt, Nümbrecht, Germany) dilution assay with a starting OD_600_ of 0.05. The assay plates were incubated on a plate shaker (Heidolph instruments, Schwabach, Germany) at 30 °C for 24h. To calculate relative growth of the bacteria, background (i.e., OD_600_ at t = 0h) was subtracted from the OD_600_ at t = 24 h and normalized to OD_600_ of an untreated control (i.e., 100% growth). Results were analyzed using GraphPad Prism software (version 6) and fitted utilizing a Gompertz function dose-response curve. The MIC was determined as the lowest concentration of nisin for which no growth was detected.

### 2.7. Radial Streak Assay

For a qualitative assessment of susceptibility and potential adaptation to nisin, a modified radial streak method was employed [62]. Fresh 2xTY agar plates were prepared and 50 µL of a nisin stock solution (250 µg/mL) spotted in the center of the plates. After drying, fresh overnight cultures of the strains (5 mL 2xTY) were streaked uniformly with an inoculation loop between the center of the nisin spot and a radial endpoint with a distance of 3.5 cm. The plates were then incubated at 30 °C for 4 days and imaged every 24 h. The radius of the inhibition zone was determined using Fiji [63].

## 3. Results

### 3.1. Distribution of Proteins Involved in Lantibiotic Resistance in the Genus Corynebacterium

**Figure 1:**
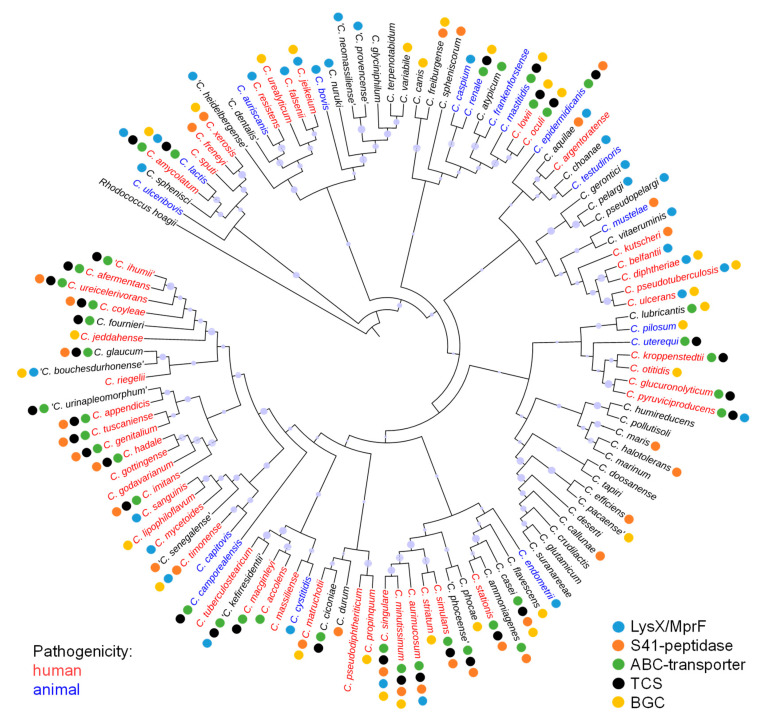
Phylogenetic arrangement of 114 *Corynebacterium* species investigated in this study. The predictions of resistance traits and bacteriocin gene clusters are indicated by colored dots behind the species name. Species that are described to be human pathogens are written in red letters, those that are animal pathogens are written in blue letters. Non-pathogenic species are depicted in black letters. Species with names that are not validly published are indicated by quotation marks. TCS = two-component system; BGC = bacteriocin gene cluster.

Resistance to bacteriocins often involves multiple factors such as cell envelope modifications, ATP-driven transporters, peptide binding lipoproteins or specific and unspecific peptidases [26]. To identify lantibiotic resistance traits of members of the genus *Corynebacterium*, publicly available genomes of 114 *Corynebacterium* species were examined by BLASTP analysis using sequences of proteins that were described to confer protection against lantibiotics (Table S2). A comprehensive overview on the species, their pathogenicity, resistance traits and bacteriocin gene clusters is presented in Figure 1.

**Figure 2:**
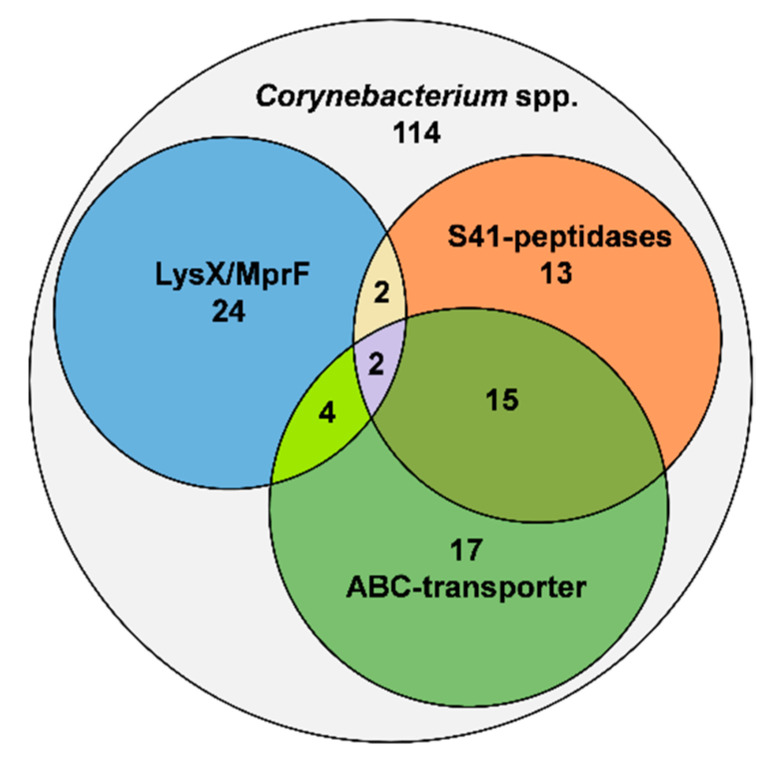
Distribution of LysX/MprF homologs (blue circle), S41-like NSR peptidases (orange) and two domain ABC-type transporters (green) in the genomes of 114 *Corynebacterium* spp.

With respect to modifications of the cell wall, the D-alanine-D-alanyl carrier protein ligase (DltA) required for D-alanylation of teichoic acids in *S. aureus* (UniProt ID P68876) and the phosphatidylglycerol lysyltransferases MprF and LysX for lysinylation of phosphatidylglycerol in *B. subtilis* and *M. tuberculosis* (UniProt IDS C0H3X7 and P9WFU7) were used as BLASTP query. No proteins with homology to DltA were identified in any of the *Corynebacterium* species. By contrast, we identified 32 species harboring homologs of LysX or MprF (Figure 2) including human pathogens like *C. diphtheriae*, *C. amycolatum*, *C. jeikeium* and *C. ulcerans* as well as pathogens of animals such as *C. bovis*, *C. pseudotuberculosis* (Figure 1). Sequence alignment of LysX from *C. bovis* and LysS from *C. lactis* with LysX from *M. tuberculosis* revealed high sequence identities of 50% and 52%, respectively (Figure S1). By contrast, the sequence identity of LysS of *C. lactis* with MprF of *B. subtilis* is only 27% (Figure S2). 

Likewise, deduced protein sequences of the *Corynebacterium* genomes were searched for homologs of the nisin-resistance protein NSR of *S. agalactiae* (UniPRot ID A0A656FZQ8) and *L. lactis* subsp. *lactis* (UniProt ID P23648). Interestingly, NSR-like peptidases were found in 32 species (Figure 2), including pathogens such as *C. minutissimum* and *C. xerosis* as well as environmental species like *C. ammoniagenes* and *C. casei* (Figure 1). Sequence alignment of the peptidases of *C. minutissimum* and *C. casei* with NSR of *S. agalactiae* showed an identity of 33% and 27%, respectively, with a conserved serine required for catalytic activity at position 236 of the *S. agalactiae* protein (Figure S3) [56]. In most cases the peptidase gene was found adjacent to the genes for a two-domain ABC transporter and a TCS.

Finally, the deduced protein sequences of the *Corynebacterium* genomes were searched for homologs of the BceAB/CprABC-type transporters of *S. agalactiae* (NsrFP; UniProt IDs X5KGL2 and A0A0E1EH51), *S. aureus* (VraDE; UniProt IDs A0A4U0CTB1 and A0A5C8XC28), *L. lactis* (NisFEG; UniProt IDs Q48635, Q48636, and Q48637) and *C. difficile* (CprABC; UniProt IDs Q18BL2, Q18BL7, and Q18BL6). Notably, we could not identify any homologs of NisE/CprB and NisG/CprC in the genus, whereas NBDs catalyzing ATP hydrolysis (i.e., NsrF, VraD, NisF, CprA) are generally highly conserved due to the abundance of different ABC-transporter families in bacteria. Queries with NsrP of *S. agalactiae* and VraE of *S. aureus* resulted in hits that did not match our e-value criteria and none of these predicted ABC-transporters was found together with one of the above identified NSR-like peptidases or a TCS. This suggests that typical CprABC- and BceAB-type transporters are not present in the analyzed genomes. Consequently, the sequence of the ABC-transporter found adjacent to the NSR-like peptidase gene of *C. casei* was used for further BLASTP analyses (W5XR13, W5XY46). By this means, we identified 38 homologous two-domain ABC transporters in the deduced protein sequences of the 114 *Corynebacterium* genomes (Figure 2). Almost all of these ABC transporter genes were located adjacent to a TCS consisting of a HK and a RR. Moreover, in 10 of the genomes including those of human pathogens like *C. macginleyi*, *C. afermentans*, *C. amycolatum*, and *C. minutissimum*, a gene for an NSR-like peptidase could be identified in the same genetic locus (Figure 1).

**Figure 3:**
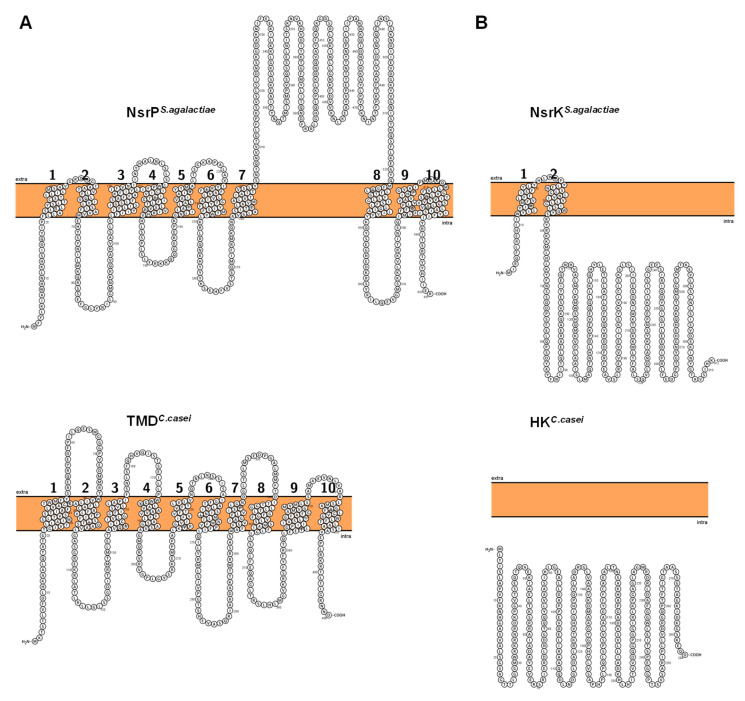
Transmembrane topology of (**A**) NsrP of *S. agalactiae* (upper left; Uniprot ID: A0A0E1EH51) and the predicted TMD in *C. casei* (lower left; Uniprot ID: W5XR13) and (**B**) HK NsrK of *S. agalactiae* (upper right; Uniprot ID: Q8DZW8) and the predicted HK in *C. casei* (lower right; UniProt ID: W5XQB7). Prediction and visualization was performed using Protter.

Each of the identified ABC-transporters contains a nucleotide-binding domain (NBD) and a single transmembrane domain (TMD). While this architecture appears similar to BceAB-type transporters, the permease subunit of e.g., *C. casei* only shares 25% sequence identity to NsrP of *S. agalactiae* (Figure S4). Furthermore, the identified TMD of *C. casei* and other *Corynebacterium* spp. are approx. 200 amino acids (aa) smaller than e.g., NsrP of *S. agalactiae*. In silico prediction of the transmembrane topology revealed ten TMHs for both proteins. However, an unusual topology was found in the analyzed *Corynebacterium* spp. permeases, which lack the large ECD between TMH 7 and 8 present in the protein of *S. agalactiae* (Figure 3A, Table S4). Also, differences in the predicted domain structure of the associated HKs compared to BceS or CrpK kinases were observed. Typically, BceS-like HKs such as e.g., NsrK of *S. agalactiae* contain two TMHs but no ECD (Figure 3B). In contrast, the two TMHs of CprK and homologs from e.g., *C. difficile* are connected by an ECD involved in sensing of the peptides (Figure S5). No THMs and thus no ECD were predicted for the HKs of *C. casei* (Figure 3B) and the other corynebacteria examined. This suggests that *Corynebacterium* species encode a novel type of lantibiotic resistance system that is different compared to hitherto described CprABC-KR and BceAB-SR systems.

### 3.2. Distribution of Bacteriocin Gene Clusters in the Genus Corynebacterium

**Figure 4:**
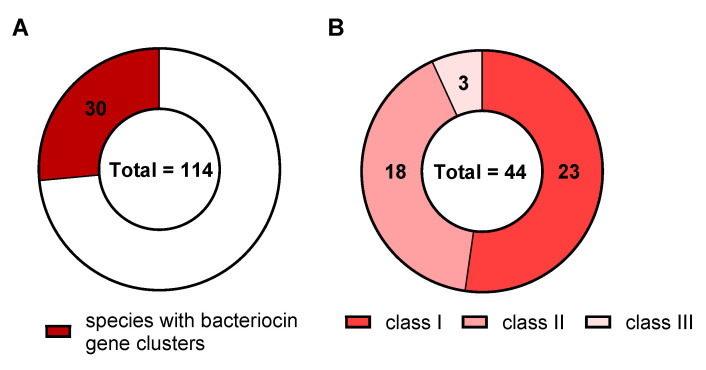
Analysis of bacteriocin gene clusters (BGC) in the genomes of 114 *Corynebacterium* spp. (**A**) Total number of *Corynebacterium* spp. with identified BGCs amongst all species analyzed. (**B**) Total number of BGCs in analyzed *Corynebacterium* spp. genomes grouped according to the three classes of bacteriocins. Analysis was performed using BAGEL4 and BLASTP.

Additionally, the presence of genes for lantibiotic production and immunity among the *Corynebacterium* species was investigated. To this end, bacteriocin gene clusters (BGCs) were searched in the publicly available genomes using BAGEL4 and BLASTP yielding 30 *Corynebacterium* species with genes potentially involved in bacteriocin biosynthesis (Figure 4A). These 30 species harbor a total of 44 BGCs (Figure 4B). Notably, BGCs for all three classes of bacteriocins were found among the analyzed genomes. BGCs for Class I bacteriocins, which comprise lantibiotics and other modified peptides, were the most prevalent class (23 BGCs), followed by BGCs for Class II (18 BGCs) and Class III (3 BGCs) bacteriocins (Figure 4B). Many of the identified Class I BGCs comprise genes for ABC transporters but none of them matched with the above described BLASTP results. To distinguish ABC-transporters involved in biosynthesis and export of bacteriocins from those involved in immunity, we also searched the BGC-positive species for the nisin exporter NisT (UniProt ID: Q03203). However, none of the ABC-transporters encoded within the BGC shared homologies to NisT. In addition, homologs of the nisin immunity protein NisI (UniProt ID: P42708) could not be identified via BLASTP analysis among the *Corynebacterium* genomes.

### 3.3. Analysis of Lantibiotic Resistance in Six Different Corynebacterium Species

Based on our in silico predictions, strains of six *Corynebacterium* species with different resistance traits were selected for further analysis (Table 2). 

**Figure 5:**
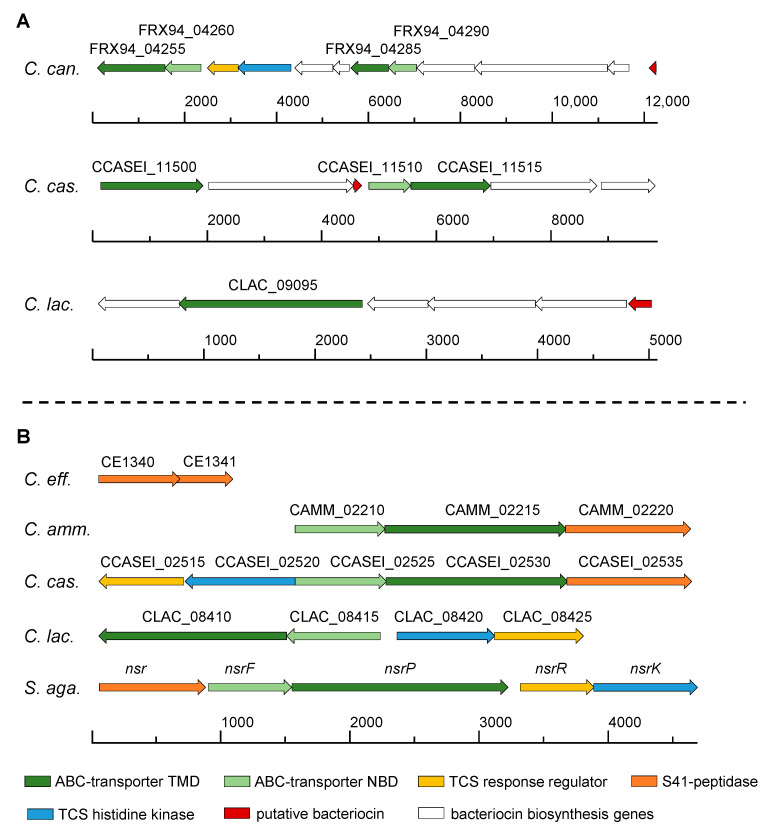
Putative BGCs (**A**) and lantibiotic resistance loci (**B**) in *C. canis* (= *C. can*.), *C. casei* (= *C. cas*.), *C. lactis* (= *C. lac*.), *C. efficiens* (= *C. eff*.), *C. ammoniagenes* (= *C. amm.*), *S. agalactiae* (= *S. aga*.). The *S. agalactiae nsr*-operon is shown for comparison. Scale bar and numbers indicate base-pair lengths. Locus tags are displayed above the respective genes.

*C. glutamicum* ATCC 13032 and *C. canis* DSM 45402 were selected as their genomes do not harbor any of the analyzed lantibiotic resistance traits despite the fact that at least *C. canis* DSM 45402 encodes a BGCs for a potential Class I bacteriocin with similarity to nisin. The genetic locus also encodes two ABC-transporters with unknown function that could be involved in immunity to the own putative bacteriocin (Figure 5A). For *C. efficiens* DSM 44549, two hits for NSR-like peptidases were obtained, which however appear to be fragmented compared to NSR of *S. agalactiae* (Figure 5B). The first fragment consists of 209 aa and has a predicted TMH from aa 21–43, while the second fragment does not have a TMH and consists of 151 aa but contains a sequence stretch TGSSGEA (aa 63–69) resembling the catalytic TASSAEM motif (Figure S6 and Figure S7) [41]. We, therefore, speculated that the peptidase might still be active. The putative lantibiotic resistance operons of *C. ammoniagenes* DSM 20306 and *C. casei* DSM 44701 both comprise genes for a two-domain ABC transporter without ECD and a gene for an NSR-like peptidase (Figure 5B). The operon of *C. casei* DSM 44701 further comprises genes for a TCS. Additionally, a BGC for a Class I bacteriocin was predicted in the genome of *C. casei* DSM 44701, comprising genes for ABC-transporters (Figure 5A). *C. lactis* RW3-42 was selected for further characterization based on its LysX-like phosphatidylglycerol lysyltransferase (Table 2) and a putative lantibiotic resistance operon with a two-domain ABC transporter and an adjacent TCS (Figure 5B). It also harbors a putative Class I BGC including an ABC-transporter with unknown function (Figure 5A). Overall, the loci encoding putative lantibiotic resistance proteins are different to the *nsr*-operon of e.g., *S. agalactiae* but the functional units (i.e., TCS and ABC transporters) are conserved.

**Figure 6:**
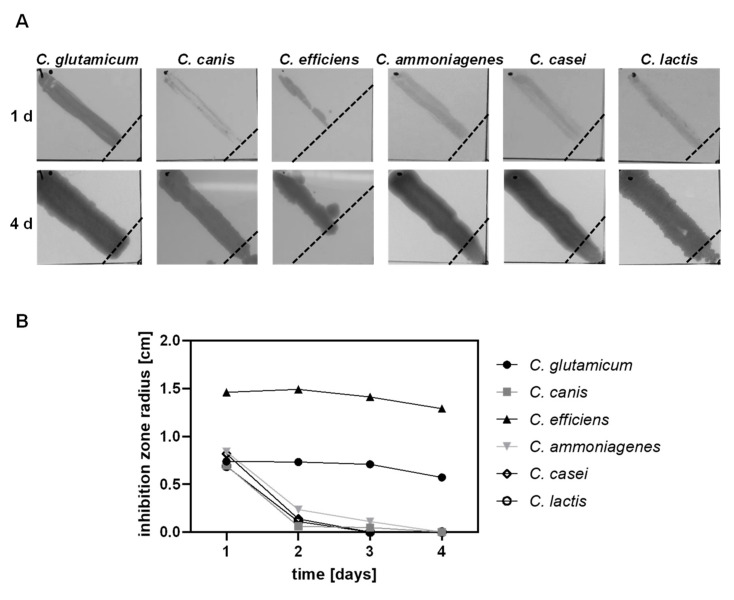
Radial streak assays of six different *Corynebacterium* species on 2xTY agar. (**A**) 50 µL of a nisin solution (0.25 mg/mL) were spotted in the middle of 2xTY agar plates. Overnight cultures of the indicated strains were streaked over a length of 3.5 cm from the black dot in the upper left corner of the image towards the center of the nisin spot. The plates were incubated for 4 days. The broken black lines indicate the boundary of growth after 24 h. (**B**) Measurements of the radius of the zone of growth inhibition from the center of the nisin spot at the indicated time after inoculation. One representative of two experiments is shown for each strain.

Susceptibility of the six selected strains to the lantibiotic nisin was assessed in a qualitative manner using a modified radial streak method. After 24 h, all six strains showed visible growth on the agar plates but inhibition of growth was observed around the spotted nisin, indicating that none of the tested strains is completely resistant against the lantibiotic (Figure 6A). The extent of the inhibition zone was comparable for *C. glutamicum* ATCC 13032, *C. canis* DSM 45402, *C. ammoniagenes* DSM 20306, *C. casei* DSM 44701 and *C. lactis* RW3-42 with a radius of 0.7–0.8 cm. In contrast, the radius of the inhibition zone for *C. efficiens* DSM 44549 was twice as long with about 1.5 cm (Figure 6B). Upon prolonged incubation, some of the strains showed growth in the zone of the nisin spot to varying degrees (Figure 6A,B). Progression of growth was particularly observed for *C. canis* DSM 45402, *C. ammoniagenes* DSM 20306, *C. casei* DSM 44701 and *C. lactis* RW3-42 of which all grew towards the center of the spotted nisin over the 4 days of incubation. However, growth of *C. glutamicum* ATCC 13032 and *C. efficiens* DSM 44549 remained at 0.6 cm and 1.3 cm of the inhibition zone radius, respectively (Figure 6B). This suggests that the other four strains may produce a factor that degrades or inactivates nisin, allowing these bacteria to increasingly grow in areas where they were previously inhibited.

Minimal inhibitory concentrations (MIC) were determined for each strain in a microtiter serial dilution assay with and without pre-cultivation in the presence of sub-lethal concentrations of nisin (Figure S8). For *C. glutamicum* ATCC 13032, a MIC of 1.25 µg/mL was determined and no induction of resistance by addition of a sub-lethal dose of nisin to the preculture was observed (Table 3). Complete inhibition of *C. canis* DSM 45402 required a 4-fold higher concentration of nisin (5.00 µg/mL) and this MIC could not be further increased by pre-induction with nisin (Table 3). *C. efficiens* DSM 44549 was highly susceptible to nisin with a MIC of 0.31 µg/mL in uninduced condition and 0.16 µg/mL in induced condition (Table 3). 

Interestingly, *C. ammoniagenes* DSM 20306 showed a MIC of 1.25 µg/mL when grown in the absence of nisin but a 4-fold increase in MIC (5.00 µg/mL) was observed when pre-cultures were grown in the presence of nisin, indicating an activation of resistance upon external stimulation with the lantibiotic (Table 3). Similarly, *C. casei* DSM 44701, which harbors a putative lantibiotic resistance operon as well as a putative BGC (Figure 5A,B), was inhibited by 0.63 µg/mL nisin and a 2-fold increase of the MIC to 1.25 µg/mL was observed when the strain was pre-cultivated with sub-lethal concentrations of the peptide (Table 3). Of all tested strains, *C. lactis* RW3-42 displayed the highest initial tolerance to nisin with a MIC of about 12.50 µg/mL without pre-induction. When nisin was added to the pre-culture 25.00 µg/mL of the peptide was required for complete inhibition of growth of this strain (Table 3). 

In summary, strains without predicted resistance proteins appear more sensitive and do not exert an inducible response to nisin, which fits to the results of our in silico analyses.

### 3.4. Heterologous Expression of Putative Lantibiotic Resistance Operons in C. glutamicum

We next sought to verify the functionality of some of the identified resistance loci via heterologous expression in a sensitive host. As *C. glutamicum* ATCC 13032 can be easily transformed and shows sensitivity towards nisin, we used this strain for our experiments. The identified operons of *C. lactis* (*nsrPFKR^Clac^*), *C. ammoniagenes* (*nsrFPX^Camm^*), and *C. casei* (*nsrRKFPX^Ccas^*) were cloned with their native promoters into the vector pJC1 (Table 1). For operons of *C. ammoniagenes* and *C. casei*, additional constructs lacking the genes for the NSR peptidase (*nsrFP^Camm^* or *nsrRKFP^Ccas^*) were cloned. After transformation of *C. glutamicum* ATCC 13032 with the respective plasmids, susceptibility to nisin was tested under the same conditions as for the original strains (Figure S9). 

Similar to the parental strain, the empty vector control strain *C. glutamicum* (pJC1) was completely inhibited by 1.25 µg/mL without any effect of pre-induction with sublethal nisin concentrations (Table 4). *C. glutamicum* (pJC_nsrPFKR*^Clac^*) harboring the TCS and ABC-transporter genes of the putative lantibiotic resistance operon of *C. lactis* did not show increased resistance to nisin (MIC: 1.25 µg/mL). Although the annotated TCS was present, no induction upon pre-cultivation in the presence of a sublethal concentration of nisin was observed (Table 4). By contrast, *C. glutamicum* (pJC_nsrRKFP*^Ccas^*), harboring the putative resistance operon of *C. casei* without the peptidase gene (*nsr*X), showed a two-fold increase in resistance to a MIC of 2.50 µg/mL. However, again no induction was observed (Table 4). Additional presence of the peptidase gene in *C. glutamicum* (pJC_nsrRKFPX*^Ccas^*) led to a MIC of 5.00 µg/mL, i.e., a four-fold increase compared to the empty vector strain (Table 4). Comparable results were obtained for the heterologous expression of the putative nisin resistance genes of *C. ammoniagenes* (Table 4).

The results of the heterologous expression experiments indicate that the predicted operons of *C. casei* and *C. ammoniagenes* indeed have a function in lantibiotic resistance, but the functionality of the ABC-transporter and the TCS remain unclear and need further investigation.

## 4. Discussion

The wide distribution of naturally occurring AMPs led to the evolution of several specific and non-specific resistance mechanisms among bacteria. Lantibiotics are one of the most interesting groups of bacteriocins as they comprise members that are used for food preservation and others that are discussed as candidates for treatment of infections with pathogenic microorganisms [19]. Thus, investigation of resistance mechanisms against lantibiotics is of high interest from a commercial and medical point of view. The aim of this study was to investigate the prevalence of lantibiotic resistance in the genus *Corynebacterium*, which comprises many important (opportunistic) pathogens, commensal bacteria of the human and animal microbiome as well as biotechnological relevant species (Figure 1).

Our in silico prediction revealed conserved resistance traits among the 114 examined *Corynebacterium* species (Figure 1). One widely distributed mechanism of resistance is the introduction of positive charges into the cell envelope to reduce attraction of cationic AMPs. For example, substitution of PG with lysine residues by enzymes such as LysX or MprF confers protection against nisin and other AMPs including defensins, which are an important component of the mammalian innate immune system. This mechanism of resistance has been shown for several pathogenic species and may represent an adaptation to their host [28,31,32]. We found that approximately one quarter of the analyzed genomes carry genes for enzymes that catalyze lysinylation of phosphatidylglycerol. This result bears some importance as frequently reported pathogens like *C. diphtheriae*, *C. ulcerans*, *C. jeikeium*, *C. bovis* and *C. pseudotuberculosis* contain LysX homologs. Similarly, D-alanylation of teichoic acids by DltA introduces positive charges into the cell envelope and was shown to mediate resistance of pathogenic *Listeria* and *Staphylococcus* species against AMPs of the innate immune system and nisin [64,65]. However, we could not identify true DltA homologs in any of the *Corynebacterium* genomes analyzed. This is in line with the fact that teichoic acids and *dlt* operons have not been described for *Corynebacterium* species so far.

A more specific type of resistance against nisin is mediated by peptidases of the NSR-type that were described to reduce the activity of nisin by proteolytic cleavage [66,67]. Indeed, approximately one quarter of the examined *Corynebacterium* genomes harbors homologs of these enzymes. NSR proteins are membrane-anchored S41 family C-terminal processing peptidases with a characteristic dyad of serine and lysine in the active site [41,68]. NSR-type peptidase genes are commonly found in bacteria that do not produce lantibiotics and are often encoded in conserved *nsr* operons together with ATP-dependent BceAB-type transporters and associated TCS. Examples of this operon structure are found in *Leuconostoc mesenteroides*, *S. agalactiae*, *Staphylococcus epidermidis* and were also predicted to be present in *L. lactis*, *C. casei* and *C. ammoniagenes* [41]. In our survey, we also detected genes for potential NSR peptidases in pathogenic *C. minutissimum* and *C. xerosis* as well as in *C. simulans* and *C. aurimucosum*, all being highly abundant members of the human skin microbiota [1]. The mechanism of NSR proteins shares similarities to LanI-type lipoproteins (e.g., NisI) of the immunity systems of lantibiotic producer strains. Here, NisI also specifically binds nisin and is assumed to act cooperatively with the ABC-type transporter NisFEG to shield the cell from the lantibiotic, however without proteolytic inactivation of its own product [69]. Interestingly, although some of the investigated *Corynebacterium* species are putative lantibiotic producers, no homolog of NisI, NisFEG or other CprABC-type transporter could be identified among them. 

It is also intriguing that the ABC-transporters identified in the putative lantibiotic resistance operons of corynebacteria appear to be structurally different from the hitherto described BceAB-type transporters like NsrFP of *S. agalactiae* or VraDE of *S. aureus*. Using the ABC transporter encoded in the putative lantibiotic resistance locus of *C. casei* for BLASTP analysis, we identified 38 homologs in the genus *Corynebacterium*. Similar to BceAB and other members of the Pep7E-family of permeases, the *Corynebacterium* transporters consist of 10 TMH and, in most cases, contain an FtsX-motif. The resistance loci in the *Corynebacterium* genomes also often comprise genes for TCS and S41-peptidases [38,41]. However, all identified transporters of *Corynebacterium* species lack the characteristic ECD between TMH 7 and 8 of the BceAB-type system. This ECD was shown to be important for substrate recognition of VraE in *S. aureus* and BceB in *B. subtilis* and directly interacts with the intramembrane histidine kinase of the TCS [36,70]. In contrast to the CprABC-type systems, the transporter and not the HK is responsible for sensing as well as ATP-dependent detoxification. In case of the newly identified system however, neither the transporters nor the histidine kinases contain the characteristic ECD, suggesting their mechanism is different from typical BceAB-RS or CprABC-RK systems. 

We tested our predictions with studies on nisin susceptibility of six *Corynebacterium* species with different predicted resistance traits. Strains like *C. glutamicum* ATCC 13032 that do not possess dedicated resistance proteins were more sensitive than others in the radial streak experiments and had lower MICs. In case of *C. efficiens* DSM 44549, the predicted NSR-fragments do not seem to provide protection against nisin as the strain had the lowest MIC of all strains tested. Overall, the *Corynebacterium* species were found to be rather sensitive to nisin compared to e.g., *S. aureus* strains that show MIC values of >128 µg/mL when the ABC- transporter VraDE is present [36]. The radial streak experiments further indicated that some of the strains (*C. casei* DSM 44701, *C. ammoniagenes* DSM 20306, *C. canis* DSM 45402 and *C. lactis* RW3-42) are able to grow into areas with high concentration of nisin over time, which could be due to degradation by an extracellular protease or delayed induction of resistance. Indeed, we could show by heterologous expression in *C. glutamicum* ATCC 13032 that the NSR-like peptidases of *C. casei* DSM 44701 and *C. ammoniagenes* DSM 20306 confer a four-fold increase in resistance to nisin, possibly by degradation. In comparison, expression of NSR in *S. agalactiae* resulted in a much higher, 20-fold increase in resistance [41]. In addition, *C. canis* DSM 45402 was described to possess extracellular trypsin activity which might lead to an inactivation of the peptide and contribute to the resistance of the strain [71]. Degradation of lantibiotics by extracellular peptidase/protease activity is of great importance for long-term topical applications of the peptide, e. g. in antimicrobial creams or as surface coatings [72]. *Corynebacterium* spp. of the skin microbiota that possess such peptidases could have a significant impact on the activity of the preparation. 

*C. casei* DSM 44701, *C. ammoniagenes* DSM 20306, and *C. lactis* RW3-42 also encode putative lantibiotic resistance loci comprising the above-mentioned ABC-transporters and TCS. Comparable systems were shown to efficiently protect the cells against lantibiotics like nisin. For example, NsrFP from *S. agalactiae* confers to a 16-fold increase in resistance when expressed in *L. lactis* NZ9000 [37]. The unusual ABC-transporter and TCS from *C. casei* DSM 44701 lead to a two-fold increase in MIC when expressed in *C. glutamicum*. This indicates that it has indeed a function in nisin resistance. By contrast, no such effect was exerted by expression of the transporter and TCS genes from *C. lactis* RW3-42 although it shares a high similarity to the *C. casei* system. One reason for the lack of functionality could be poor expression from the native promoter of the *C. lactis* operons in *C. glutamicum*. As the results for the here tested ABC-transporters and TCS remain ambiguous, further investigations are needed to elucidate their exact functions. In addition, and in contrast to the original wild type strains, we could not observe an induction effect for the recombinant *C. glutamicum* strains after incubation with sublethal concentrations of nisin. The above discussed absence of an ECD in the ABC-transporter and the HK of the analyzed *Corynebacterium* species might indicate a missing component in our setup.

Similar to induction of resistance, induction of lantibiotic production occurs via TCS as it is the case for the well described NisRK of *L. lactis* [40]. While we could not identify true nisin producers among the *Corynebacterium* species, some of the analyzed genomes revealed putative BGCs with peptides that share a high similarity to the model bacteriocin. We speculated that these species might display some sort of cross immunity by encoding immunity/resistance genes in the endogenous BGCs. Among the tested strains, *C. casei* DSM 44701, *C. canis* DSM 45402 and *C. lactis* RW3-42 possess BGCs for Class I bacteriocins that comprise genes for ABC-transporters. In this respect, *C. canis* DSM 45402, which does not possess any other resistance trait than its putative BGC, indeed showed a significantly higher resistance to nisin compared to e.g., *C. glutamicum* ATCC 13032. However, as we could not specifically identify immunity transporters and cross-immunity is regarded as rare, it remains speculative whether the predicted BGC has an influence on the susceptibility to nisin. In case of *C. lactis* RW34-2 the high innate and inducible nisin resistance might more likely go back to its predicted LysX homolog. Changes of the cell surface charge by incorporation of L-PG by LysX was suggested as a highly effective mechanism of resistance against cationic peptides and is often described for pathogenic species [26]. 

In summary, our study presents a comprehensive analysis of lantibiotic resistance genes in the genus *Corynebacterium* and suggests the presence of operons that encode for peptidases and transporters with an unusual structure and potentially novel mechanism. These findings may be relevant for biotechnological and medical applications as the genus comprises various frequently and infrequently reported pathogens as well as species important for industry.

## Figures and Tables

**Table 1:** Bacterial strains and plasmids used in this study.

Species	Strain	Reference
*Corynebacterium glutamicum*	ATCC 13032	ATCC ^a^
*Corynebacterium ammoniagenes*	DSM 20306	[42]
*Corynebacterium lactis*	RW3-42	[43]
*Corynebacterium efficiens*	DSM 44549	[44]
*Corynebacterium casei*	DSM 44701	[45]
*Corynebacterium canis*	DSM 45402	[46]
**Plasmid**	**Characteristics**	
pJC1	*E. coli/C. glutamicum* shuttle vector;	[47]
pJC_nsrPFKR*^Clac^*	pJC1-derivative with *nsr*-like operon of *C. lactis* RW3-42	This study
pJC_nsrRKFP*^Ccas^*	pJC1-derivative with *nsr*-like operons of *C. casei* DSM 44701 lacking the peptidase gene *nsrX*	This study
pJC_nsrRKFPX*^Ccas^*	pJC1-derivative with *nsr*-like operons of *C. casei* DSM 44701;	This study
pJC_nsrFP*^Camm^*	pJC1-derivative with *nsr*-like operon of *C. ammoniagenes* DSM 20306 lacking the peptidase gene *nsrX*;	This study
pJC_nsrFPX*^Camm^*	pJC1-derivative with *nsr*-like operon of *C. ammoniagenes* DSM 20306;	This study

^a^ American Type Culture Collection.

**Table 2:** *Corynebacterium* strains chosen for further characterization and their resistance traits.

Species	Strain	BGC	LanI	CprABC	BceAB	NSR	HK	RR	LysX
*C. glutamicum*	ATCC 13032	-	-	-	-	-	-	-	-
*C. ammoniagenes*	DSM 20306	-	-	-	+	+	-	-	-
*C. lactis*	RW3-42	+	-	-	+	-	+	+	+
*C. efficiens*	DSM 44549	-	-	-	-	(+) ^a^	-	-	-
*C. casei*	DSM 44701	+	-	-	+	+	+	+	-
*C. canis*	DSM 45402	+	-	-	-	-	-	-	-

^a^ present in two independent orfs.

**Table 3:** Minimal inhibitory concentrations (MIC) of various *Corynebacterium* species strains determined in the experiments shown in Figure S8 with (induced) or without (uninduced) preincubation in medium containing sublethal concentrations of nisin.

Strain	MIC [µg/mL]	
	Uninduced	Induced
*C. glutamicum* ATCC 13032	1.25	1.25
*C. canis* DSM 45402	5.00	5.00
*C. efficiens* DSM 44549	0.31	0.16
*C. ammoniagenes* DSM 20306	1.25	5.00
*C. casei* DSM 44701	0.63	1.25
*C. lactis* RW3-42	12.50	25.00

**Table 4:** Minimal inhibitory concentrations (MIC) of recombinant *Corynebacterium glutamicum* strains expressing different resistance loci determined in the experiments shown in Figure S9 with (induced) or without (uninduced) preincubation in medium containing sublethal concentrations of nisin.

Strain	MIC [µg/mL]	
	Uninduced	Induced
*C. glutamicum* ATCC 13032/pJC1	1.25	1.25
*C. glutamicum* ATCC 13032/pJC1_*nsrPFKR^Clac^*	1.25	1.25
*C. glutamicum* ATCC 13032/pJC1_*nsrRKFP^Ccas^*	2.50	2.50
*C. glutamicum* ATCC 13032/pJC1_*nsrRKFPX^Ccas^*	5.00	5.00
*C. glutamicum* ATCC 13032/pJC1_*nsrFP^Camm^*	1.25	1.25
*C. glutamicum* ATCC 13032/pJC1_*nsrFPX^Camm^*	5.00	5.00

## Data Availability

Data is contained within the article and supplementary material.

## Supplementary Materials

The following are available online at https://www.mdpi.com/2076-2607/9/3/646/s1, Table S1: List of *Corynebacterium* genomes used for in silico prediction of resistance proteins and bacteriocin gene clusters., Table S2: Proteins and enzymes conferring resistance against lantibiotics that were used for the in silico predictions in this study, Table S3: Oligonucleotides used in this study, Figure S1: MAFFT sequence alignment of LysX from *M. tuberculosis*, LysX from *C. bovis* and LysS from *C. lactis*, Figure S2: MAFFT sequence alignment of MprF from *B. subtilis* and LysS from *C. lactis*, Figure S3: MAFFT sequence alignment of NSR-like (S41-) peptidases from *S. agalactiae*, *C. casei* and *C. minutissimum*, Figure S4: MAFFT sequence alignment of NsrP from *S. agalactiae* and *C. casei*, Table S4: Predicted ABC-transporters using the TMD from *C. casei*, Figure S5: Prediction of TM-, inner- and outer-segments of the histidine kinase CprK of *C. difficile*, Figure S6: MAFFT sequence alignment of NSR from *S. agalactiae* and *C. efficiens*, Figure S7: MAFFT sequence alignment of NSR from *S. agalactiae* and *C. efficiens*, Figure S8: Susceptibility of selected *Corynebacterium* species to nisin following growth in the presence or absence of a sublethal concentration of nisin, Figure S9: Susceptibility to nisin of *C. glutamicum* strains harboring *nsr*-operon encoding plasmids following growth in the presence or absence of a sublethal concentration of nisin.
