# Supplementary material for: In Silico Prediction and Analysis of Unusual Lantibiotic Resistance Operons in the Genus Corynebacterium
Source: Microorganisms. 2021 Mar 19;9(3):646. doi: 10.3390/microorganisms9030646 (PMC8003753; doi:10.3390/microorganisms9030646)
Supplement: Supplementary file 1 [file microorganisms-09-00646-s001.pdf]

microorganisms

Supplementary data

In silico prediction and analysis of unusual lantibiotic resistance operons in the genus *Corynebacterium*

Oliver Goldbeck[1,*], Dominik Weixler[1], Bernhard J. Eikmanns[1], and Christian U. Riedel[1,*]

[1]Institute of Microbiology and Biotechnology, Ulm University
* Correspondence: oliver.goldbeck@uni-ulm.de; christian.riedel@uni-ulm.de

Table S1. List of *Corynebacterium* genomes used for *in silico* prediction of resistance proteins and bacteriocin gene clusters.

Species	Strain	Source	Accession Number [1]
C. accolens	ATCC 49726	human cervix	GCF_000146485.1
C. afermentans	DSM 44280	human blood culture	GCF_900156035.1
C. ammoniagenes	DSM 20306	soil	GCF_000164115.1
C. amycolatum	SK46	human skin	GCF_000173655.1
C. appendicis	CIP 107643	patient with appendicitis	GCF_900156665.1
C. aquilae	DSM 44791	choanae of golden eagle	GCF_001941445.1
C. argentoratense	DSM 44202	human throat	GCF_000590555.1
C. atypicum	R2070	human clinical specimen	GCF_000732945.1
C. aurimucosum	ATCC 700975	human clinical specimen	GCF_000174695.1
C. auriscanis	CIP 106629	dog ear infection	GCF_000767255.1
C. belfantii	FRC0043	pseudomembrane	GCF_900205605.1
C. bouchesdurhonense	SN14	human stool	GCF_900078305.2
C. bovis	DSM 20582	bovine milk	GCF_000183325.1
C. callunae	DSM 20147	unclear	GCF_000344785.1
C. camporealensis	DSM 44610	sheep with mastitis	GCF_000980815.1
C. canis	CCUG 58627	infected wound	GCF_007859215.1
C. capitovis	DSM 44611	infected head of a sheep	GCF_000372085.1
C. casei	LMG S-19264	smear cheese	GCF_000550785.1
C. caspium	DSM 44850	caspian seal, penis	GCF_000379705.1
C. choanis	200CH	northern bald ibis	GCF_003813965.1
C. ciconiae	DSM 44920	stork trachea	GCF_000372385.1
C. coyleae	DSM 44184	human blood	GCF_900105505.1
C. crudilactis	JZ16	bovine milk	GCF_001643015.1
C. cystitidis	DSM 20524	urine of cow	GCF_900111265.1
C. dentalis	Marseille-P4122	human dental plaque	GCF_900232865.1
C. deserti	GIMN1.010	desert sand	GCF_001277995.1
C. diphtheriae	DSM 43989	human sample	GCF_001913255.1
C. doosanense	CAU 212	wastewater	GCF_000372245.1
C. durum	F0235	human sputum	GCF_000318135.1
C. efficiens	YS-314	soil	GCF_000011305.1
C. endometrii	LMM-1653	bovine endometritis	GCF_004795735.1
C. epidermidicanis	DSM 45586	skin of a dog	GCF_001021025.1
C. falsenii	355_CFAL	human blood	GCF_001054945.1
C. flavescens	OJ8	cheese	GCF_001941465.1

Table S1. Continued

Species	Strain	Source	Accession Number
C. fournierii	Marseille-P2948	human vaginal sample	GCF_900176865.1
C. frankenforstense	DSM 45800	milk tank of a dairy farm	GCF_001941485.1
C. freiburgense	DSM 45254	infected wound	GCF_000428805.1
C. freneyi	DNF00450	pus of a toe	GCF_000758965.1
C. genitalium	ATCC 33030	clinical isolate	GCF_000143825.1
C. geronticis	W8	northern bald ibis	GCF_003813985.1
C. glaucum	DSM 30827	clinical material	GCF_002287505.1
C. glucuronolyticum	DSM 44120	human ejaculate	GCF_900176155.1
C. glutamicum	ATCC 13032	soil	GCF_000011325.1
C. glyciniphilum	AJ 3170	putrefied bananas	GCF_000626675.1
C. godavarianum	LMG 29598	river water	GCF_007559235.1
C. gottingense	DSM 103494	human blood	GCF_003693265.1
C. hadale	NBT06-6	deep sea water	GCF_002273005.1
C. halotolerans	YIM 70093	saline soil	GCF_000341345.1
C. heidelbergense	DSM 104638	Egyptian geese	GCF_003285565.1
C. humireducens	NBRC 106098	microbial fuel cell	GCF_000819445.1
C. ihumii	GD7	human faeces	GCF_000403725.1
C. imitans	DSM 44264	human throat	GCF_000739455.1
C. jeddahense	JCB	human faeces	GCF_000577555.1
C. jeikeium	ATCC 43734	human blood	GCF_000163435.1
C. kefirresidentii	SB	kefir grains	GCF_002154655.1
C. kroppenstedtii	DSM 44385	human sputum	GCF_000023145.1
C. kutscheri	DSM 20755	lung abscess of mouse	GCF_000980835.1
C. lactis	RW2-5	raw cow milk	GCF_001274895.1
C. lipophiloflavum	DSM 44291	vaginal swab	GCF_000159635.1
C. lowii	NML 130206	human eye	GCF_001412085.1
C. lubricantis	DSM 45231	coolant lubricant	GCF_000379425.1
C. macginleyi	CCUG 32361	human corneal ulcer	GCF_003688935.1
C. marinum	DSM 44953	coastal sediment	GCF_000835165.1
C. maris	DSM 45190	coral mucus	GCF_000442645.1
C. massiliense	DSM 45435	osteoarticular infection	GCF_000420605.1
C. mastitidis	DSM 44356	sheep mastitis	GCF_000375365.1
C. matruchotii	ATCC 33806	oral calculus	GCF_000158635.1
C. minutissimum	ATCC 23348	erythrasma	GCF_000805675.1
C. mustelae	DSM 45274	lung tissue of a ferret	GCF_001020985.1
C. mycetoides	DSM 20632	tropicaloid ulcer	GCF_900103625.1
C. neomassiliense	Marseille-P3888	human stool	GCF_900626215.1
C. nuruki	S6-4	fermentation starter	GCF_000213935.1
C. oculi	NML 130210	human eye	GCF_001412105.1
C. otitidis	ATCC 51513	human ear fluid	GCF_000296405.1
C. pacaense	Marseille-P2417	human stool	GCF_900169525.1
C. pelargi	136/3	stork trachea	GCF_004114895.1
C. phocae	M408/89/1	common seal	GCF_001941565.1
C. phoceense	MC1	human urine	GCF_900092335.1
C. pilosum	DSM 20521	urine of cow	GCF_000373805.1
C. pollutisoli	VDS11	soil	GCF_900177745.1
C. propinquum	DSM 44285	human respiratory tract	GCF_000375525.1
C. provencense	SN15	human stool samples	GCF_900049755.1
C. pseudodiphtheriticum	DSM 44287	human throat	GCF_000688415.1

Table S1. Continued

Species	Strain	Source	Accession Number
C. pseudopelargi	812CH	northern bald Ibis	GCF_003814005.1
C. pseudotuberculosis	ATCC 19410	gland of sheep	GCF_002155265.1
C. pyruviciproducens	ATCC BAA-1742	human abscess	GCF_000411375.1
C. renale	DSM 20688	cow	GCF_002563965.1
C. resistens	DSM 45100	human blood	GCF_000177535.2
C. riegelii	PUDD_83A45	human urine	GCF_001263755.1
C. sanguinis	CCUG 58655	human blood	GCF_007641235.1
C. senegalense	Marseille-P4329	human	GCF_900411315.1
C. simulans	PES1	human lymph node	GCF_001586215.1
C. singulare	IBS B52218	human semen	GCF_000833575.1
C. sphenisci	DSM 44792	penguin	GCF_001941505.1
C. spheniscorum	J11	cloacae of penguin	GCF_900113445.1
C. sputi	DSM 45148	patient with pneumonia	GCF_000427865.1
C. stationis	DSM 20302	sea water	GCF_001941345.1
C. striatum	ATCC 6940	unclear	GCF_000159135.1
C. suranareeae	N24	starling's faeces	GCF_002355155.1
C. tapiri	LMG 28165	tonsil of a tapir	GCF_006334925.1
C. terpenotabidum	Y-11	soil	GCF_000418365.1
C. testudinoris	DSM 44614	tortoise wound	GCF_001021045.1
C. timonense	DSM 45434	human blood	GCF_900105305.1
C. tuberculostearicum	SK141	human bone marrow	GCF_000175635.1
C. tuscaniense	DNF00037	human blood	GCF_000759055.1
C. ulcerans	BR-AD22	human throat	GCF_000215665.1
C. ulceribovis	DSM 45146	ulcer of a cattle	GCF_000372445.1
C. urealyticum	DSM 7109	human bladder stone	GCF_000069945.1
C. ureicelerivorans	IMMIB RIV-2301	blood culture	GCF_000747315.1
C. urinapleomorphum	Marseille-P2799	urine sample	GCF_900155535.1
C. uterequi	DSM 45634	horse uterus	GCF_001021065.1
C. variabile	DSM 44702	food	GCF_000179395.2
C. vitaeruminis	DSM 20294	cow rumen	GCF_000550805.1
C. xerosis	ATCC 373	ear discharge	GCF_000988235.1

[1] retrieved from the NCBI database (version of April 2020).

Table S2: Proteins and enzymes conferring resistance against lantibiotics that were used for the *in silico* predictions in this study.

Species	Protein	Uniprot code	Resistance type	Function
Lactococcus lactis subsp. *lactis*	NisI	P42708	Lipoprotein	Lipoprotein
	NisF	Q48635		NBD
	NisE	Q48636	CprABC	TMD
	NisG	Q48637		TMD
	NSR	P23648	Peptidase	Peptidase
	NisT	Q03203	none	Export of nisin (biosynthesis)
Clostridioides difficile	CprA	Q18BL2		NBD
	CprB	Q18BL7	CprABC	TMD
	CprC	Q18BL6		TMD
	CprK	Q18BL5	Two component system	Histidine kinase
Staphylococcus aureus	VraD	A0A4U0CTB1	BceAB	NBD
	VraE	A0A5C8XC28		TMD
Corynebacterium casei[1]	AHI19089.1	W5XR13	BceAB	TMD
	AHI19088.1	W5XY46		NBD
	AHI19090.1	W5XQX4	Peptidase	Peptidase
	AHI19087.1	W5XQB7	Two component system	Histidine kinase
Streptococcus agalactiae	NsrP	A0A0E1EH51	BceAB	TMD
	NsrF	X5KGL2		NBD
	NSR	A0A656FZQ8	Peptidase	Peptidase
	NsrK	Q8DZW8	Two component system	Histidine kinase
Bacillus subtilis	MprF	C0H3X7	Cell membrane modification	PG-lysinylation
Mycobacterium tuberculosis	LysX	P9WFU7	Cell membrane modification	PG-lysinylation
Staphylococcus aureus	DltA	P68876	Cell wall modification	D-alanine--D-alanyl ligase

[1] Not experimentally verified; *in silico* prediction by Khosa et al., 2013. NBD = nucleotide binding domain; TMD = transmembrane domain; PG = peptidoglycan.

Table S3: Oligonucleotides used in this study.

Primer	Sequence (5′ → 3′)	Purpose
nsrFP_fwd (lactis)	GATCAGCGACGCCGCAGGGGAGGGTTAATTAATCTCAGCCTC	pJC_nsrPFKR^{Clac}
nsrFP_rev (lactis)	GCACTACTACGAGAAGGTAGTGGGCACTG	
nsrRK_fwd (lactis)	CTACCTTCTCGTAGTAGTGCTAGGTCTAC	
nsrRK_rev (lactis)	GTTGTTGCCATTGCTGCAGGTCTAGATGACCTAATAAACTACCTTG	
nsrFP_fwd (ammo)	GATCAGCGACGCCGCAGGGGGATCCTGTTGTGCGGCAG	pJC_nsrFP^{Camm}
nsrFP_rev (ammo)	GTTGTTGCCATTGCTGCAGGTTAAGCATTGTCTCGTCCAATC	
nsrFP-X_fwd (ammo)	GATCAGCGACGCCGCAGGGGGATCCTGTTGTGCGGCAG	pJC_nsrFPX^{Camm}
nsrFP-X_rev (ammo)	GTTGTTGCCATTGCTGCAGGCTACCGGCAACCCTGCTTTG	
nsrKR_fwd (casei)	GATCAGCGACGCCGCAGGGGGCGTGGTCTTTGTGTACTTCTCAAAG	pJC_nsrRKFP^{Ccas}
nsrKR_rev (casei)	AATTCCCTTGAGCATGGCTGGCAGTGCC	
nsrFP_fwd (casei)	CAGCCATGCTCAAGGGAATTTCGCGCTC	
nsrFP_rev (casei)	GTTGTTGCCATTGCTGCAGGCGAAGAGGGATAGAAGAATCTTC	
nsrFP-KR-X_fwd (casei)	GATCAGCGACGCCGCAGGGGGCGTGGTCTTTGTGTACTTCTC	pJC_nsrRKFPX^{Ccas}
nsrFP-KR-X_rev (casei)	GTTGTTGCCATTGCTGCAGGCTCAACACGCTACCGGCAAC	
pJC1_fw	CAAAGGGGAAGCCTCCAAT	Control of pJC1 inserts
pJC1_rev	CAATAAACCAGCCAGCCGG	
pJC1-1 seq1	CAATGTCGTTGGCAAAGGTGG	
pJC1-1 seq2	GTCATTGTCGAAACTGACCAAG	
pJC1-1 seq3	GCGCGCTGGAGCCTGAAGCTGAG	
pJC1-1 seq4	CATTTCACTTAGCGGAGCG	
pJC1-1 seq5	GTAGTGGTGCATGTGCCCTAC	
pJC1-2 seq1	CACGCTGACGATTGGACGAGAC	
pJC1-2 seq2	GCAGCGCTTCCATGGCTTTC	
pJC1-3 seq1	CAATGTCGTTGGCAAAGGTGG	
pJC1-5 seq1	GTCATTGTCGAAACTGACCAAG	

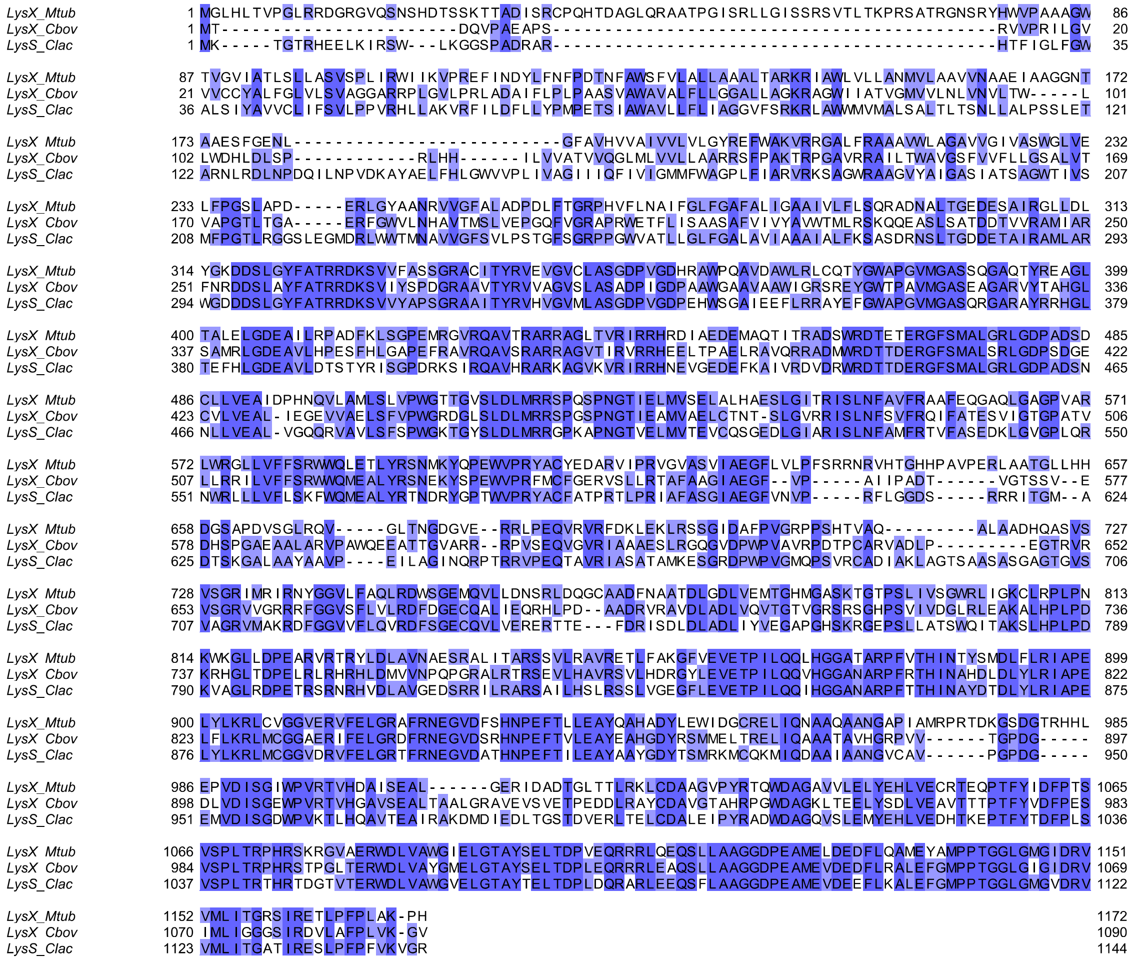

Figure S1. MAFFT sequence alignment of LysX from *M. tuberculosis* (= *LysX_Mtub*), LysX from *C. bovis* (= *LysX_Cbov*) and LysS from *C. lactis* (= *LysS_Clac*).

```
MprF_Bsub      1 MLIKKN-----------------------------ALSILKIVF-------PIAVLL--------FVIYQSKKELTNLSFKRTLMVING--   45
LysS_Clac      1 MKTGTRHEELKIRSWLKGGSPADRARHTFIGLFGWALSIYAVVCLIFSVLPPVRHLLAKVRFILDFLLYPMPE--TSIAWAVLLFLIAGGV   89

MprF_Bsub     46 LERTDLFMLVLIGLLAVAAMSLYDYVLKYSLRLSITNGKVFR-------VSWIANSFNNVLGFGG-----LAGVGLR-----MMFY-----  114
LysS_Clac     90 FSRKRLAWWM---VMALSALTLTSNLL--ALPSSLETARNLRDLNPDQILNPVDKAYAELFHLGWVVPLIVAGIIIQFIVIGMMFWAGPLF  175

MprF_Bsub    115 ----KEHTKDHKALVKGI--------AWLTSSMLLGLS-------------VFSIFVAARVLPVDEVIHEKPWLWAVVIG-FALILPLSLA  179
LysS_Clac    176 IARVRKSAGWRAAGVYAIGASIATSAGWTIVSMFPGTLRGGSLEGMDRLWWTMNAVVGFSVLPSTGFSGRPPGWVATLLGLFGALAVIAAA  266

MprF_Bsub    180 VSKIK---DRKA--GDEENADKVKNPIFAYIGASVVEWLMAGTVIYFA-------LFAMGIHADIRYVFGVFVIAAIGGMISLVPGGFGSF  258
LysS_Clac    267 IALFKSASDRNSLTGDDETA----------IRAMLARWGDDDSLGYFATRRDKSVVYAPSGRAAITYRVHVGVMLASGDPVGDPEHWSGAI  347

MprF_Bsub    259 DLLFLLGMEQLGYHQEAIVTSIVLYRLAYSFIPFILGLFFAAGDLTENTMKRLETNPRIAPAIETTNVLLVVQRA----VLVRILQ----G  341
LysS_Clac    348 E-EFLRRAYEFGWAPGVMGAS---QRGARAYRRHGLTEFHLGDEAVLDTSTYRISGP------DRKSIRQAVHRARKAGVKVRIRRHNEVG  428

MprF_Bsub    342 SLSLIVFVAGL-------------IVLASVSLPIDRLTVIPHI----PRPALLLFNGLSLSSALILLILPIELYKRTKRSYTMAITALVGG  415
LysS_Clac    429 EDEFKAIVRDVDRWRDTTDERGFSMALGRLGDPADSNNLLVEALVGQQRVAVLSFSPWGKTG------YSLDLMRRGPKAPNGTVELMVTE  513

MprF_Bsub    416 FVFSFLKGLNISAIFVLPMIIVLLVLLKKQFVRE--------QASYTLGQLIFAVALFTV-ALFNYNLIAGFIWDRMKKVLRHEYFV--HS  495
LysS_Clac    514 VCQS---GEDLG----IARISLNFAMFRTVFASEDKLGVGPLQRNWRL-LLVFLSKFWQMEALYRTNDRYGPTW-----VPRYACFATPRT  591

MprF_Bsub    496 TSHITHATIMA--IIIVPLFF--------------------------LIFTVVYHKRTKPIGEKADPERLAAFLNEKGGNALSHLG-----  553
LysS_Clac    592 LPRIAFASGIAEGFVNVPRFLGGDSRRRITGMADTSKGALAAYAAVPEILAGINQRPTRRVPEQTAVRIASATAMKESGRDPWPVGMQPSV  682

MprF_Bsub    554 -------FLGDKRFYFSSDGNALLLFGKI-ARR------LVVLGDPSGQRESFPLVLEEFLNEAHQKGFSVLFYQIEREDMA--LYHDFGY  628
LysS_Clac    683 RCADIAKLAGTSAASASGAGTGVSVAGRVMAKRDFGGVVFLQVRDFSGECQV--LVERERTTE---------FDRISDLDLADLIYVEGAP  762

MprF_Bsub    629 NFFKLGEEAYVDLNTFTLTGK-------KKAGLRAINNRFEREEYTFHVDHPPFSDA----------------------FLEELKQISDEW  690
LysS_Clac    763 GHSKRGEPSLL-ATSWQITAKSLHPLPDKVAGLRDPETRSRNR----HVDLAVGEDSRRILRARSAILHSLRSSLVGEGFLEVETPILQQI  848

MprF_Bsub    691 LGSKKEKGFS--LGFFDPS-YLQKAPIAYMK--------------------------NAEGEIV-AFAN----------VMPMYQEGEISV  741
LysS_Clac    849 HGGANARPFTTHINAYDTDLYLRIAPELYLKRLMCGGVDRVFELGRTFRNEGVDATHNPEFTILEAYAAYGDYTSMRKMCQKMIQDAAIAA  939

MprF_Bsub    742 -----------DLMRYRGDAP-------------------------------NGIMDALFI-------------RMFLWAKEEGC------  771
LysS_Clac    940 NGVCAVPGPDGEMVDISGDWPVKTLHQAVTEAIRAKDMDIEDLTGSTDVERLTELCDALEIPYRADWDAGQVSLEMYEHLVEDHTKEPTFY 1030

MprF_Bsub    772 TSFNMGMAPLANV----GTAFTSFWSERFAAVIFNNVRYMYSFSGL------RAFKEKYK-------PE--------WRGKYLAYRKNRSL  837
LysS_Clac   1031 TDFPLSVSPLTRTHRTDGTV-----TERWDLVAW-GVELGTAYTELTDPLDQRARLEEQSFLAAGGDPEAMEVDEEFLKALEFGMPPTGGL 1115

MprF_Bsub    838 SVTMFLVTRLI-GKSKKDSV---------                                                                856
LysS_Clac   1116 GMGVDRVVMLITGATIRESLPFPFVKVGR                                                               1144
```

Figure S2. MAFFT sequence alignment of MprF from *B. subtilis* (= *MprF_Bsub*) and LysS from *C. lactis* (= *LysS_Clac*).

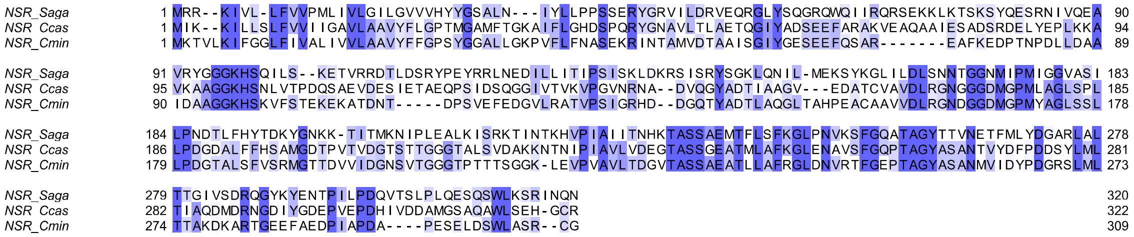

Figure S3. MAFFT sequence alignment of NSR-like (S41-) peptidases from *S. agalactiae* (= *NSR_Saga*), *C. casei* (= *NSR_Ccas*) and *C. minutissimum* (= *NSR_Cmin*).

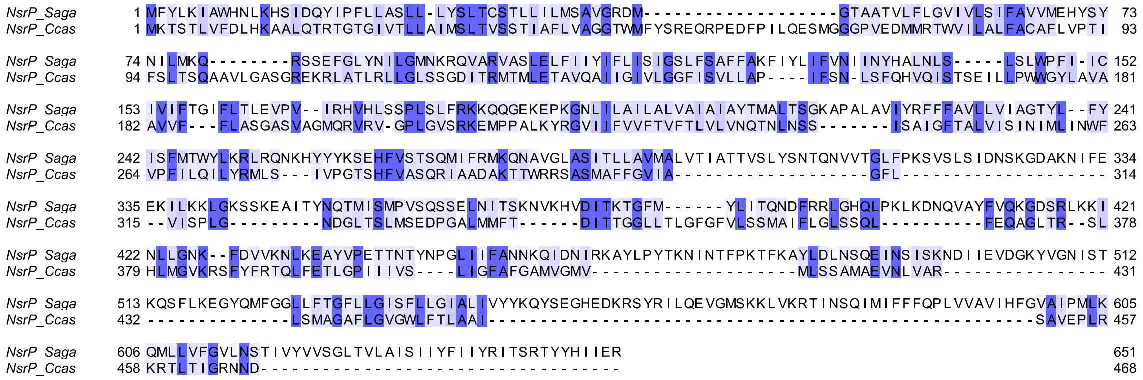

Figure S4. MAFFT sequence alignment of NsrP from *S. agalactiae* (= *NsrP_Saga*) and *C. casei* (= *NsrP_Ccas*).

Table S4. Predicted ABC-transporters using the TMD from *C. casei* (Uniprot ID: W5XR13) as BLASTP query. Co-occurrence of the ABC-transporters with TCS and peptidase genes was analyzed. TM-prediction was performed using TMHMMv2.0.

Species	e-value	NBD[1] accession number	e-value	TMD[2] accession number	TCS[3]	S41 peptidase	ECD[4]	TMH[5]
C. casei	4e-169	AHI19088.1	0.0	AHI19089.1	+	-	-	10
C. stationis	3e-150	WP_066792391.1	0.0	WP_066792392.1	+	+	-	10
C. ammoniagenes	1e-149	APT81782.1	0.0	APT81783.1	-	+	-	10
C. stationis	-	WP_066840035.1	0.0	WP_066840034.1	+	+	-	10
C. lowii	4e-111	WP_055177214.1	2e-163	WP_055177216.1	+	-	-	10
C. phoceense	9e-110	WP_141628946.1	4e-159	WP_141628945.1	+	+	-	10
C. aurimucosum	3e-108	ACP33843.1	4e-158	ACP33842.1	+	+	-	10
C. kefirresidentii	2e-110	WP_086588220.1	2e-157	WP_086588219.1	+	-	-	10
C. minutissimum	2e-108	WP_115023669.1	7e-157	WP_115023666.1	+	+	-	10
C. minutissimum	-	WP_12801832.1	2e-156	WP_046647193.1	+	+	-	10
C. minutissimum	3e-108	WP_039676650.1	7e-153	WP_039676648.1	+	+	-	10
C. singulare	4e-109	WP_144793994.1	9e-151	WP_144793991.1	+	+	-	10
C. simulans	2e-113	WP_061924749.1	1e-149	WP_061924744.1	+	-	-	10
C. macginleyi	1e-110	WP_121911496.1	2e-141	WP_121911497.1	+	-	-	10
C. macginleyi	1e-110	WP_121952825.1	2e-141	WP_121927657.1	+	-	-	10
C. mastitidis	2e-103	WP_018117593.1	6e-140	WP_018117594.1	+	-	-	10
C. mastitidis	4e-103	WP_101172955.1	1e-138	WP_101172956.1	+	-	-	10
C. epidermidicanis	2e-104	WP_144413531.1	2e-130	WP_047241170.1	+	-	-	10
C. oculi	1e-106	WP_055121515.1	7e-125	WP_082422102.1	+	-	-	10
C. lubricantis	4e-105	WP_018298083.1	9e-125	WP_026196388.1	-	-	-	10
C. camporealensis	2e-126	AKE38696.1	8e-121	AKE38697.1	+	-	-	10
C. accolens	9e-110	EFM42783.1	9e-116	EFM42803.1	-	-	-	9
C. camporealensis	-	AVH87978.1	7e-115	AVH87979.1	+	-	-	10
C. camporealensis	1e-126	WP_035107392.1	1e-109	WP_144407160.1	+	-	-	10
C. camporealensis	1e-126	WP_035107392.1	1e-95	WP_105360296.1	+	-	-	8
C. kroppenstedtii	3e-92	ACR17280.1	5e-90	ACR17281.1	+	-	-	10
C. amycolatum	1e-103	EEB62915.1	7e-85	EEB62962.1	+	-	-	10
C. uterequi	6e-95	WP_047258977.1	3e-83	WP_047258978.1	+	-	-	10
C. lactis	3e-103	ALA67737.1	1e-75	ALA68591.1	+	-	-	10
C. ciconiae	8e-91	WP_018019232.1	6e-60	WP_018019231.1	+	-	-	10
C. tuscaniense	5e-93	KGF21162.1	1e-59	KGF21163.1	+	-	-	10
C. glaucum	4e-97	WP_154839513.1	2e-59	WP_154839515.1	+	-	-	10
C. coyleae	-	WP_070422001.1	1e-58	WP_101732792.1	+	-	-	10
C. fournierii	5e-97	WP_085956990.1	3e-55	WP_085956989.1	+	-	-	10
C. coyleae	1e-97	WP_167616151.1	5e-55	WP_167616153.1	+	-	-	10
C. ihumii	-	WP_034996836.1	1e-54	WP_158293355.1	+	-	-	10
C. glaucum	6e-97	WP_095658995.1	3e-53	WP_095658996.1	+	-	-	10
C. hadale	4e-94	WP_095275266.1	2e-51	WP_095275265.1	+	-	-	10
C. hadale	5e-96	WP_095535955.1	5e-51	WP_095535879.1	+	-	-	10
C. appendicis	3e-94	SIS38884.1	4e-50	SIS38879.1	+	-	-	10
C. coyleae	2e-97	WP_101740780.1	9e-50	WP_101740779.1	+	-	-	10
C. pyruviciproducens	1e-77	EPD68012.1	1e-49	EPD68013.1	+	-	-	10
C. afermentans	1e-97	OAA16131.1	2e-49	OAA16132.1	+	-	-	10
C. ureicelerivorans	5e-96	WP_038609086.1	2e-49	WP_038609089.1	+	-	-	10

Table S4. Continued

Species	e-value	NBD accession number	e-value	TMD accession number	TCS	S41 peptidase	ECD	TMH
C. hadale	4e-94	WP_095275266.1	2e-51	WP_095275265.1	+	-	-	10
C. hadale	5e-96	WP_095535955.1	5e-51	WP_095535879.1	+	-	-	10
C. appendicis	3e-94	SIS38884.1	4e-50	SIS38879.1	+	-	-	10
C. coyleae	2e-97	WP_101740780.1	9e-50	WP_101740779.1	+	-	-	10
C. pyruviciproducens	1e-77	EPD68012.1	1e-49	EPD68013.1	+	-	-	10
C. afermentans	1e-97	OAA16131.1	2e-49	OAA16132.1	+	-	-	10
C. ureicelerivorans	5e-96	WP_038609086.1	2e-49	WP_038609089.1	+	-	-	10
C. hadale	1e-95	WP_095538045.1	3e-49	WP_095538018.1	+	-	-	10
C. hadale	-	WP_095548127.1	4e-49	WP_095548091.1	+	-	-	10
C. glucuronolyticum	6e-88	EEI62477.1	1e-48	EEI62478.1	+	-	-	10
C. urinapleomorphum	1e-93	WP_087116979.1	1e-48	WP_087116980.1	+	-	-	10
C. coyleae	2e-97	WP_167594486.1	4e-48	WP_092101153.1	+	-	-	10
C. hadale	1e-95	WP_095554574.1	5e-48	WP_095554501.1	+	-	-	10
C. genitalium	2e-90	EFK55196.1	2e-47	EFK55195.1	+	-	-	10
C. imitans	2e-95	WP_038587675.1	3e-47	WP_038587678.1	+	-	-	10
C. renale	2e-86	WP_111726555.1	1e-45	WP_111726557.1	+	-	-	10
C. atypicum	-	AIG64238.1	9e-27	AIG64237.1	-	-	-	8

[1] nucleotide binding domain
[2] transmembrane domain
[3] two-component system
[4] extracellular domain
[5] transmembrane helices

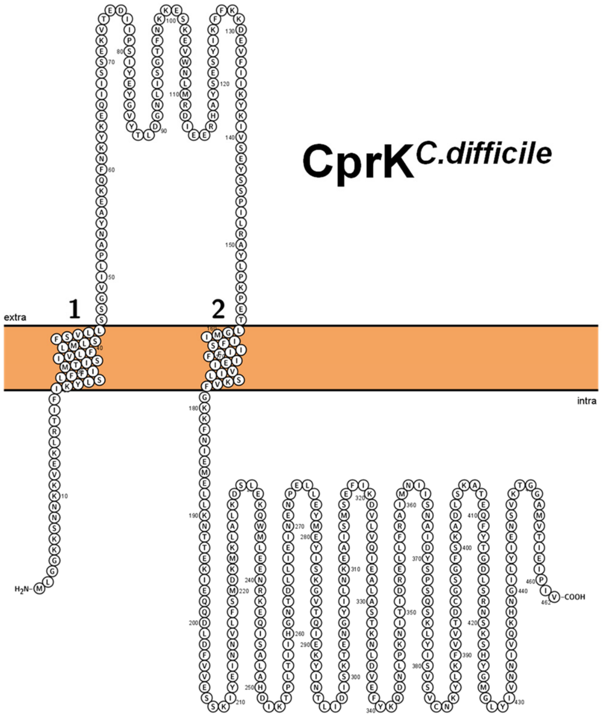

Figure S5. Prediction of TM-, inner- and outer-segments of the histidine kinase CprK (Uniprot ID: Q18BL5) of *C. difficile*.

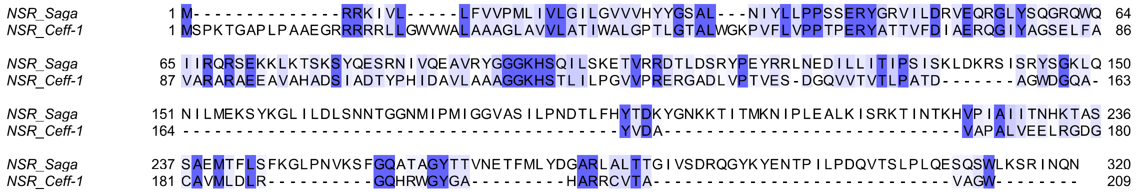

Figure S6. MAFFT sequence alignment of NSR from *S. agalactiae* (*NSR_Saga*) and *C. efficiens* (*NSR_Ceff-1*).

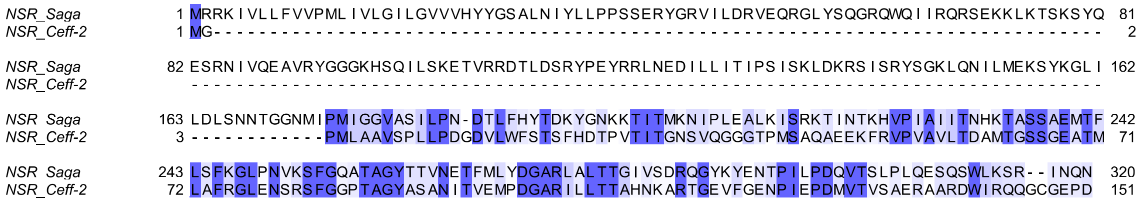

Figure S7. MAFFT sequence alignment of NSR from *S. agalactiae* (*NSR_Saga*) and *C. efficiens* (*NSR_Ceff-2*).

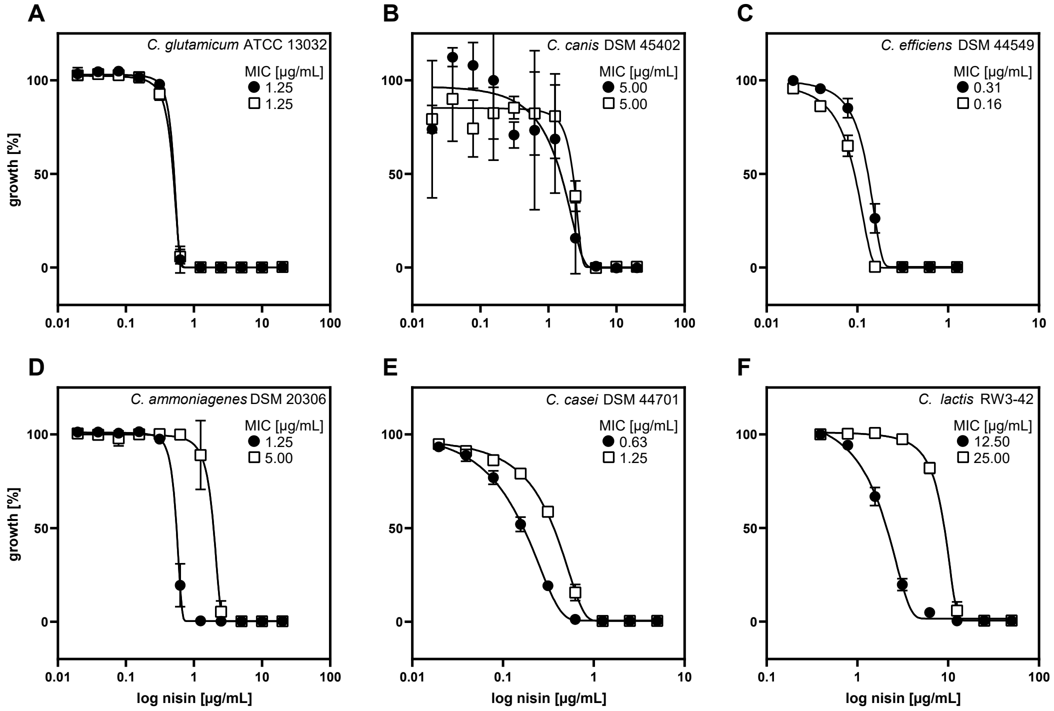

Figure S8. Susceptibility of selected *Corynebacterium* species to nisin following growth in the presence (open squares) or absence (black circles) of a sublethal concentration of nisin. (A) *C. glutamicum* ATCC 13032. (B) *C. canis* DSM 45402. (C) *C. efficiens* DSM 44549. (D) *C. ammoniagenes* DSM 20306. (E) *C. casei* DSM 44701. (F) *C. lactis* RW3-42. Values are mean ± standard deviation of n=3 independent cultivations. Regression curves were calculated utilizing a Gompertz function.

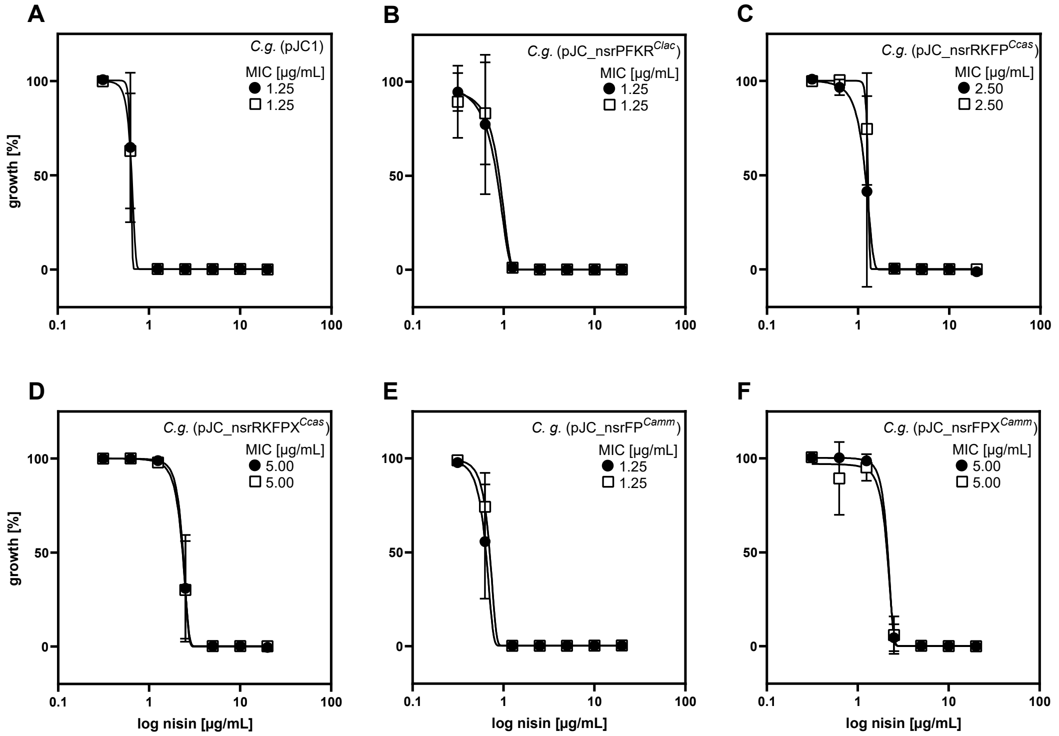

Figure S9. Susceptibility to nisin of *C. glutamicum* strains harboring *nsr*-operon encoding plasmids following growth in the presence (open squares) or absence (black circles) of a sublethal concentration of nisin. (A) *C. glutamicum* (pJC1). (B) *C. glutamicum* (pJC_nsrPFKR^{Clac}). (C) *C. glutamicum* (pJC_nsrRKFP^{Ccas}). (D) *C. glutamicum* (pJC_nsrRKFPX^{Ccas}). (E) *C. glutamicum* (pJC_nsrFP^{Camm}). (F) *C. glutamicum* (pJC_nsrFPX^{Camm}). Values are mean ± standard deviation of n=3 independent cultivations. Regression curves were calculated utilizing a Gompertz function.
